# Expanding access to HIV testing through Canadian community pharmacies: findings from the APPROACH study

**DOI:** 10.1186/s12889-020-08719-0

**Published:** 2020-05-07

**Authors:** Deborah V. Kelly, Jason Kielly, Christine Hughes, Jacqueline Gahagan, Shabnam Asghari, Stephanie Hancock, Kimberley Burt, Petra Smyczek, Carmen Charlton, Hai Nguyen

**Affiliations:** 1 School of Pharmacy, Memorial University of Newfoundland, 75 Tiffany Court, St. John’s, NL A1A 0L1 Canada; 2 Faculty of Pharmacy and Pharmaceutical Sciences, University of Alberta, Edmonton, AB Canada; 3 School of Health and Human Performance, Dalhousie University, Halifax, NS Canada; 4 Discipline of Family Medicine, Memorial University of Newfoundland, St. John’s, NL Canada; 5 Memorial University of Newfoundland, St. John’s, Canada; 6 Eastern Health, St. John’s, NL Canada; 7 Alberta Health Services, Edmonton, AB Canada; 8 Public Health Laboratory, Edmonton, AB Canada

**Keywords:** HIV, Point-of-care testing, Rapid HIV testing, Community pharmacy, Pharmacist, Implementation, Public health

## Abstract

**Background:**

There is a need for acceptable and feasible HIV testing options to ensure people living with HIV know their status so they can access care. Pharmacist-provided HIV point-of-care testing (POCT) may overcome testing barriers, including privacy concerns, testing wait times, and improve accessibility. In the APPROACH study, we aimed to develop and assess an HIV POCT program in community pharmacies for future scale up and evaluation. This paper describes the program uptake, participant and pharmacist experiences, and implementation factors.

**Methods:**

A pharmacist-provided HIV POCT program was offered in 4 pharmacies in two Canadian provinces. A mixed methods design incorporated self-report questionnaire data, participant telephone interviews, pharmacist focus groups, workload analysis, and situational analysis to assess the uptake, acceptability and feasibility of the HIV POCT program.

**Results:**

Over the 6-month pilot, 123 HIV tests were performed. One new case of HIV was identified; this participant was linked with confirmatory testing and HIV care. Participants were predominantly male (76%), with a mean age of 35 years. This was the first HIV test for 27% participants, and 75% were at moderate to very high risk of undiagnosed HIV infection, by Denver HIV Risk Score. Questionnaires and telephone interviews showed participants were very satisfied with the program; 99% agreed HIV POCT should be routinely offered in pharmacies and 78% were willing to pay for the service. Participants felt the pharmacy was convenient, discreet, and that the pharmacist was supportive and provided education about how to reduce their future risk. Pharmacists felt prepared, confident, and expressed professional satisfaction with offering HIV POCT. Community and public health supports, clear linkage to care plans to refer participants with positive HIV POCT results, and provision of counselling tools were important enabling factors for the program. Pharmacist remuneration, integration with existing healthcare systems, and support for ongoing promotion of HIV POCT availability in pharmacies were identified as needs for future scale-up and sustainability.

**Conclusions:**

A successful model of pharmacy-based POCT, including linkage to care, was developed. Further research is needed to determine the effectiveness and cost-effectiveness of this approach in finding new diagnoses and linking them with care.

**Trial registration:**

Retrospectively registered with clinicaltrials.gov (NCT03210701) on July 6, 2017.

## Background

A key component toward achieving the United Nations 90–90-90 goals aimed at ending the HIV epidemic worldwide depends heavily on achieving the first 90 target; that is, 90% of people living with HIV are diagnosed [1]. This is crucial as over 50% of new HIV infections are believed to occur as the result of transmission from those who are unaware of their HIV infection, and because those who do know their HIV status are more likely to reduce behaviours that can lead to HIV transmission [2, 3]. Yet barriers to HIV testing are well recognized - stigma, fear of disclosure, concerns over privacy, low perceived risk of infection, lack of access to testing, delays for appointment times, and lack of testing outside business hours, to name a few [4–6]. Innovative approaches that make HIV testing more accessible and acceptable to those at risk are urgently needed.

Point-of-care testing (POCT) for HIV has been shown to improve access to and uptake of HIV testing in areas where healthcare resources are limited and among high risk populations [7–12]. Advantages of HIV POCT include ease of administration and return of results within minutes, making the test ideal for use outside of traditional healthcare settings, such as community testing sites and within pharmacies. Pharmacy-based testing programs are appealing due to the widespread availability and ease of access to pharmacies in nearly every community. Pharmacists’ scope of practice in North America includes providing immunizations and POCT for a variety of indications in many jurisdictions. Pharmacists are a trusted source for education and counselling making them well-positioned to offer HIV POCT services, including linkages to care with other health professionals and programs for follow up care.

The APPROACH^1^ study was designed as a pilot to develop and assess the implementation of a novel pharmacy-based HIV testing model in two Canadian provinces. Our goal was to develop a novel program using existing pharmacy and community resources that would be feasible and could eventually be scaled up to allow for future assessment of effectiveness, sustainability and cost-effectiveness. Importantly, the program had to include linkage to care plans to ensure that participants with a reactive HIV POCT result received confirmatory testing, counselling, and connection to HIV care. This paper describes the main results of the APPROACH study, including the uptake and outcomes associated with the development of the HIV POCT program in pharmacies, acceptability of the testing program as experienced by clients and pharmacists, and an analysis of factors integral to the success of implementing a pharmacy-based screening program for HIV.

## Methods

The APPROACH study was funded by a grant from the Canadian Institute of Health Research. We used a type-2 hybrid implementation-effectiveness study design [13] to develop and assess the feasibility and acceptability of a pharmacist-provided HIV POCT program in two Canadian provinces: Newfoundland and Labrador (NL) and Alberta (AB). A mixed methods design incorporated self-report questionnaire data, telephone interviews of participants, pharmacist focus groups, analysis of workload logs, and situational analysis to assess the uptake, acceptability and feasibility of the HIV POCT program.

### Development of the HIV POCT program

A full description of our study methods has been published [14]. Advisory Committees were established in each province to provide input into the program design, linkages to care pathways, and promotional plans to invite the public to participate in the new HIV POCT program. Each Advisory Committee was comprised of key provincial stakeholders deemed critical to the success of the program, including public health and policy makers, pharmacists and managers, HIV-experienced health workers, community representatives from organizations who work with individuals at risk of HIV, and individuals themselves with lived experience.

One urban (population >  100, 000) and one rural (population <  100, 000) pharmacy in each province were selected to participate in the study. Three of the pharmacies were large chain pharmacies and were open 7 days per week; two pharmacies were open during the evenings. One pharmacy was independently owned and operated with daytime hours Monday to Friday and limited weekend hours. The two pharmacies in urban settings were centrally located and provided opioid substitution therapy services. Testing was offered by appointment at all four sites, and two pharmacies offered walk-in testing during advertised hours. All pharmacies had a private room for counselling and performing POCT. Each site had to have at least one trained pharmacist tester in order to participate in the pilot study.

Participating pharmacists completed a training program that consisted of an online self-study module, in-person training day, in-pharmacy competency assessment, and proficiency assessment. Details of the training program have been published [14]. The training program covered the consent process, pre- and post-test counselling, how to administer the HIV POCT and interpret the test result. Pharmacists were provided with tools to aid in counselling, contact information for linkages to care and client support services in their area.

### Study procedures and population

Participants were not actively recruited by the pharmacists but rather the availability of HIV POCT through participating pharmacies was promoted through news media, social media, posters displayed throughout each community, as well as through active promotion by community partners and organizations who worked with at-risk individuals. Paid advertising through Grindr, a geosocial networking and online dating application geared toward gay, bi, trans, and queer people [15], was also used for a limited period during the study in NL.

Adults aged 18 years or older were eligible to participate if they provided written informed consent, and had an active healthcare number (for linkage to care purposes). Participants who indicated they had been previously diagnosed with HIV infection or had a previous positive HIV test were not eligible.

**Fig. 1:**
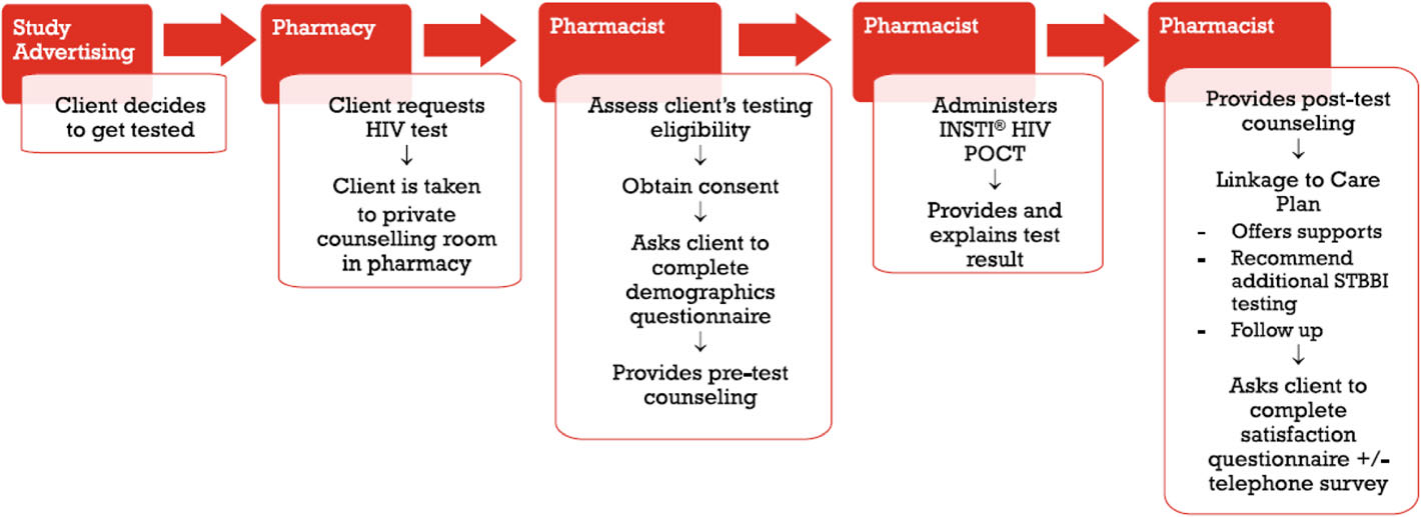
HIV POCT process in community pharmacies

Potential participants could request HIV testing at one of the participating pharmacies during advertised testing hours or by appointment. Potential participants were taken to a private counselling room where they met with the pharmacist who screened participants for eligibility and completed the testing process (Fig. 1). The INSTI® HIV-1/HIV-2 rapid antibody test (BioLytical Laboratories, Richmond, BC, Canada) was administered free of charge using a finger-prick blood sample. Results were read within 60 s by the pharmacist and the participant was advised of their result. Participants received pre and post-test counselling based on national testing guidelines [16], including advice regarding additional sexually transmitted and bloodborne infection (STBBI) testing. In the event of a reactive test result, the pharmacist provided the participant with a bloodwork requisition to obtain confirmatory HIV testing and additional supports and counselling were offered. Confirmatory test results were received by a designated health provider (physician or nurse practitioner) per the linkage to care plan in each province.

### Data collection

Pharmacists recorded the date, participant study ID, test lot number, test result, and time to complete each test in a work log. Participants were asked to complete two de-identified (pre and post testing) questionnaires, which were blinded from the pharmacist. The first questionnaire included demographic data (age, gender, ethnicity, relationship status, highest education attained, and income level) as well as information about HIV risk factors and previous HIV testing history. The second questionnaire assessed perception of the testing experience including factors which influenced their decision to be tested at the pharmacy and whether they would have sought HIV testing elsewhere if not at the pharmacy. Satisfaction questions were aimed to differentiate influences of the pharmacy testing environment from the characteristics of the HIV POCT.

Participants were also offered the choice of participating in a telephone interview with a research assistant (RA) at a later time to permit a more in-depth exploration of their testing experience and their motivation for choosing to be tested at the pharmacy. Clients who agreed were offered the option to have their name entered into a draw for grocery gift certificates in appreciation for their time. Semi-structured interviews were conducted by two RAs, one in each province, to explore questions about their testing experience at the pharmacy including why they chose to get tested, what they liked and did not like about their testing experience, and willingness to pay for testing.

To assess the pharmacists’ perceptions of the testing program, pharmacist testers attended a focus group with members of the research team in each province after the end of the study. Pharmacists were asked about how well the training and program resources supported them to offer the program, as well as questions aimed at understanding the impact of offering the testing program on their workload, the work environment/staff response, sense of professional role and satisfaction. Discussions also explored their fidelity to the testing process, what modifications to the program were made and why, and the implications for scalability and sustainability of the pharmacist testing program.

### Data analysis

Participant characteristics, responses to satisfaction measures on the participant questionnaire, and pharmacist time to offer HIV POCT were analyzed using descriptive statistics. Pre-testing questionnaire data was used to calculate a Denver HIV Risk Score [17, 18] for each participant, as a means to predict their probability of having an undiagnosed HIV infection. Analysis of variance (ANOVA) was used to assess differences in participants’ Denver HIV Risk Scores between provinces, and urban versus rural testing sites, and multivariate analysis of variance (MANOVA) was used to assess differences in participant satisfaction with the testing experience based on province, urban/rural testing site, sex, and history of prior HIV testing. Fisher’s Exact tests were used to assess whether history of prior HIV testing depended on province or urban/rural centre for testing.

Qualitative data were analysed using a thematic analysis approach [19]. Interview transcripts and extensive field notes were included for analysis from the participant interviews and pharmacist focus groups. Transcripts from participant interviews were coded using an open or emergent scheme where codes were developed and modified throughout the coding process. Descriptive codes were assigned to identify recurring concepts, and sub-themes were then identified and grouped into significant broader themes. Coding was performed independently by two RAs to improve inter-rater reliability. After completing coding individually, the RAs met several times to discuss the coding process and propose themes, with disagreements discussed in detail until consensus was reached. Emerging themes were grouped and triangulated with the members of the research team (DK, JK, CH) to determine final themes and important considerations emerging from the data. Themes from the pharmacist focus groups were considered according to the COM-B model [20] to understand behaviour change, which considers opportunities and challenges in the context of professional capability, opportunity, and motivation to offer the HIV POCT program.

### Ethical considerations

This study was approved by the human research ethics boards in each province. All participants and pharmacists signed study consent forms in keeping with the ethics protocol. Identifiable participant information was stored separately from trial data, and participants were encouraged to provide pseudonyms or false names if they wished for the purposes of being contacted by the RA for their telephone interviews. No identifying information was kept by the study team.

## Results

A total of 123 tests were conducted between February and September 2017, with nearly 10% of tests completed in rural communities (8/89 in NL and 3/34 in AB). Of the 123 tests performed, there was one reactive result which was subsequently confirmed to be a new HIV diagnosis. This participant received confirmatory HIV testing and was successfully linked with the provincial HIV program for follow up care within 72 h of the reactive HIV POCT result in the pharmacy. The remaining 122 test results were non-reactive; there were no indeterminate or invalid test results.

On average, pharmacists spent 30.4 min (range 10–50, SD 6.95) completing the testing process. This included time spent explaining the study and consenting the participant, providing pre-test counselling, administering the test and explaining the result, post-test counselling, and referring the participant for additional STBBI testing as indicated.

### Characteristics of participants

Demographic characteristics of the participants are described in Table 1. Participants were primarily male (75.6%), Caucasian (79.7%), and reported being single (34.1%) or casually dating (31.7%). The mean age of participants was 34.8 years (range 18–97, SD 12.9), and 27.3% reported this was their first HIV test experience.

**Table 1:** Participant self-reported characteristics

	NL *n (%)* *n* = 89	AB *n (%)* *n* = 34	Overall *n (%)* *n* = 123
*Gender*			
Male	66 (74.2)	27 (79.4)	93 (75.6)
Female	21 (23.6)	7 (20.6)	28 (22.8)
Trans person	0	0	0
Other	2 (2.2)	0	2 (1.6)
Mean Age, years (range)	34.8 (18–97)	34.7 (21–53)	34.8 (18–97)
*Ethnicity*			
Caucasian/White	77 (86.5)	21 (61.8)	98 (79.7)
African, Caribbean, or Black	2 (2.2)	6 (17.6)	8 (6.5)
Indigenous	0	2 (5.9)	2 (1.6)
Asian	8 (9)	4 (11.8)	12 (9.8)
Other	2 (2.2)	1 (2.9)	3 (2.4)
*Testing Location*			
Urban (> 100, 000 population)	81 (91)	31 (91.2)	112 (91.1)
Rural (< 100, 000 population)	8 (9)	3 (8.8)	11 (8.9)
*Relationship status*			
Single	30 (33.7)	12 (35.3)	42 (34.1)
Casually dating	28 (31.5)	11 (32.4)	39 (31.7)
Married, Common-Law or Committed Relationship	24 (27)	10 (29.4)	34 (27.6)
Separated, Divorced or Widowed (SDW)	5 (5.6)	1 (2.9)	6 (4.9)
SDW and Casually Dating	2 (2.2)	0	2 (1.6)
*Employment/Student Status (n = 122)*			
Working full-time	47 (53.4)	26 (76.5)	76 (62.3)
Working part-time	2 (2.3)	2 (5.9)	4 (3.3)
Student	15 (17)	2 (5.9)	17 (13.9)
Retired or Receiving Disability Support	8 (9.1)	1 (2.9)	9 (7.4)
Not employed	9 (10.2)	3 (8.8)	12 (9.8)
Working and Student	7 (8)	0	7 (5.7)
*Highest Level of Education*			
Some high school (< grade 12)	2 (2.2)	0	2 (1.6)
High school diploma or equivalent	18 (20.2)	7 (20.6)	25 (20.3)
Some university or college	17 (19.1)	4 (11.8)	21 (17.1)
College, Trade or Technical School diploma/certificate	23 (25.8)	7 (20.6)	30 (24.4)
University degree	37 (41.6)	16 (47.1)	53 (43.1)
*Annual Income*			
< $20,000	27 (30.3)	5 (14.7)	32 (26)
$20,000 to $39,999	20 (22.5)	5 (14.7)	25 (20.3)
$40,000 to $59,999	19 (21.3)	7 (20.6)	26 (21.1)
$60,000 to $79,999	9 (10.1)	2 (5.9)	11 (8.9)
$80,000 to $99,999	4 (4.5)	10 (29.4)	14 (11.4)
> $100,000	10 (11.2)	5 (14.7)	15 (12.2)
*Tested for HIV previously (n = 121)*			
No	25 (28.7)	8 (23.5)	33 (27.3)
Yes	60 (69)	26 (76.5)	86 (71.1)
Unsure	2 (2.3)	0	2 (1.7)

Table 2 describes the participants’ self-identified HIV risk behaviours. The most common risk behaviour was men who had sex with men (MSM), and 75% of participants tested had a calculated Denver HIV Risk Score greater than 30 points, suggesting these participants had a moderate to very high risk for undiagnosed HIV infection [17, 18]. There was no difference in Denver HIV Risk Score between provinces (*P*-value for two-way ANOVA = 0.980) or whether participants were tested at urban or rural sites (*P*-value for two-way ANOVA = 0.231) in each province. Among participants who provided information about their prior HIV testing history, the majority (68.8%) of individuals for whom this was their first HIV test were at moderate to very high risk of undiagnosed HIV infection, based on Denver HIV Risk Score. A Fisher’s Exact test showed participants’ history of a prior HIV test did not depend on their province (*p* = 0.517) or whether they were tested at an urban or rural site (*p* = 1.000).

**Table 2:** Participant self-reported risk behaviours

Exposure Category	No. (%)^a^
Men who have sex with men	56 (47.1)
History of intravenous drug use	6 (5.1)
Previously exchanged sex for money or drugs	8 (6.8)
Prior blood transfusion	2 (1.7)
Denver HIV Risk Score ≥ 30 points^b^	89 (75.4)

^a^ Not all patients answered all questions; percentage calculated based on number of respondents for each question

^b^ Patients who score 30 points or greater on the Denver HIV Risk Score are considered at increased risk of undiagnosed HIV infection and should be offered routine screening [17, 18]

Participants reported travelling an average of 23 min (range 2–180, SD 25.2) to get to the pharmacy where they were tested. At the rural NL site, the average travel distance was longer (43.1 min in NL; range 5–150, SD 50.7, versus 8.3 min in AB; range 5–15, SD 5.8). Notably, only three tests were conducted at the rural AB site; the single trained pharmacist at this pharmacy left their employment so this study site was terminated early.

### Participant perceptions of the testing experience

Results from the post-testing questionnaires indicated that participants felt a high degree of satisfaction with their HIV POCT experience (Table 3). Four-way MANOVA tests indicated there were no differences in participant satisfaction based on their province (*p* = 0.266), urban/rural testing site (*p* = 0.842), sex (*p* = 0.595), or history of prior HIV testing (*p* = 0.489), and all possible interactions between these four factors produced non-significant results (all *p* values > 0.05).

**Table 3:** Participant perceptions of their HIV POCT experience

Post-test Questionnaire Item (with a percentage continuous response scale)	N	Mean	Median
1. How comfortable did you feel today getting your HIV test at the pharmacy?	121	88.66	95.00
2. How confident are you that the pharmacist did a good job of administering your HIV test today?	122	96.93	100
3. How likely are you to also get tested for other infections (e.g., hepatitis C or syphilis), based on the pharmacist’s advice today?	121	83.14	95.00
4. How important was each of the following factors in helping you decide to get an HIV test at this pharmacy today?			
a. We keep your test results private (confidential)	120	89.42	97.50
b. The HIV test uses a finger-prick sample (instead of a blood test)	120	80.35	95.00
c. Your test results are available to you immediately (within minutes)	120	96.08	100
d. We have a private room for testing and consultation	120	94.87	100
e. The testing is free	120	88.78	98.00
f. You did not need to make an appointment	86^c^	91.22	100
5. Would you be willing to pay for an HIV test at a pharmacy if it were offered as a regular service?			
Yes →	122	78.69% (96/122)	
How much would you be willing to pay? $________^d^	92	$29.43	$20.00
No →	122	21.31% (26/122)	
6. How likely are you to recommend to your friends that they get tested for HIV at a pharmacy?	120	92.80	100
7. Do you think that HIV testing should be offered through pharmacies?			
Yes →	122	99.2% (121/122)	
No →	122	0.8% (1/122)	

^c^ Only NL respondents are included as AB pharmacies offered testing on a per-appointment basis only

^d^ Based on responses from those clients who expressed willingness to pay for the service

Participants reported feeling comfortable getting tested at the pharmacy, a high degree of confidence in the pharmacist performing the test, and a high likelihood of pursuing testing for additional STBBI based on the pharmacist’s advice. (Table 3) The most important factors that influenced participants’ decisions to pursue an HIV test at the pharmacy included the ability to receive their results immediately and availability of a private room for testing at the pharmacy. In NL, participants could choose to make an appointment or drop-in during advertised testing hours, and the ability to get tested without having to make an appointment was cited as an important factor. Over 78% of participants indicated they would be willing to pay for an HIV test at the pharmacy, and the median amount they would be willing to pay was $20.00 (Canadian). There was nearly unanimous agreement that HIV testing should be routinely offered through pharmacies.

Thirty-six (29.3%) participants completed the telephone interview, which comprised the qualitative assessment of testing experience. Major reasons cited by respondents for choosing to get tested included uncertainty of their HIV status, recent travel, new relationships, or other recently recognized risk behaviours. Participants who indicated they hadn’t thought about being tested previously said they were attracted by the option of getting tested at a pharmacy specifically, and of those with a regular testing routine, the convenience, accessibility and immediate results provided by the HIV POCT were appealing.

Participants were overwhelmingly positive in their approval for the HIV POCT programs. Key themes relating to aspects that participants found appealing were the convenience and accessibility of both the pharmacy and of the HIV POCT itself. The program was felt to be convenient, expeditious, and participants liked receiving their results immediately. The pharmacy environment was felt to provide a degree of normalcy and anonymity around the testing process, yet the consultation room where the testing was conducted provided privacy and contributed to a comfortable experience. Positive pharmacist behaviours (e.g. kindness, non-judgemental, comforting/supportive) were highlighted as factors they liked most about the program. Participants indicated they learned new information from the pharmacist, including how to reduce future risk (e.g. pre-exposure prophylaxis, condom education) and several participants expressed their intent to get tested for additional STBBIs as result of the pharmacist’s advice. Participants cited a variety of emotional or cognitive reasons for preferring to be tested at the pharmacy. For example, the pharmacy is a familiar venue that contributed to their sense of ease, and many people felt that getting tested at the pharmacy was less stigmatizing than going to a sexual health clinic or their doctor. Overall there were few negative comments about the program. Some felt that the pipette used to collect the finger-prick blood sample was uncomfortable or not convenient, and it was suggested that there should have been better advertising to increase awareness of the program. Participant quotes regarding their pharmacy testing experience are in Table 4.

**Table 4:** Quotes related to the participant testing experience

**Liked Accessibility/Convenience of pharmacy**	
“Having it at the drugstore helps a lot because it’s more convenient for young people and just to drop in.”	
“I know when I went to make an appointment [at the sexual health clinic] – a couple of times I’ve been on a 2 or 3 week wait. So with this it was get in, get out, perfect. “	
**Liked Accessibility/Convenience of POCT**	
“The pharmacy thing was fantastic because it was so quick. If I could change anything it would be the traditional routes of getting tested and having to wait a week for the results to come back. It would be better if it could be the 2-minute kind of thing.”	
**Liked Confidentiality/Privacy/Anonymity**	
“I liked the fact that you’re going into the consultation room – the sign on the door just said consultation room. You don’t know if someone’s going in there getting their methadone, you don’t know if someone’s a new diabetic, you don’t know if someone’s going in there showing them how to use their needles, you don’t know what’s going on behind that door. And there’s nobody else there listening, no windows, nobody looking at you. It was private. You’re not intimidated sitting there waiting. But it was confidential, no one knew what I was going in for.”	
“It was completely…I’m using the word anonymous but there must be another word…more discrete. Well, if I had went to a major hospital, I’m in a small town, everybody knows everybody, I know all their staff at the hospital, they know me, they see me every day if it’s my day up there.”	
**Liked Positive pharmacist behaviours**	
“Yes, the [pharmacist] that did mine was obviously very well trained and knew what she was talking about. She was deadly. And she was like super…she put me at ease, we’ll say. She was great.”	
**Liked Positive emotional/cognitive aspects**	
“The first thing is, where you’re in a [pharmacy], it feels like you’re just out shopping. You don’t feel weird. When I go into a hospital, I hate it – or even a medical centre you know, like an [hospitalbased] medical centre, I get a funny feeling. I don’t like that either. You feel like you’re dropping in at [the pharmacy] and it’s not stressful, because it’s already a stressful situation.”	
“I think when it’s sort of like, tucked away and you can only go here [sexual health clinic] to get it [HIV testing] done, then it’s like ‘oh that person is going there because they have this’ – I think it [having testing in pharmacy] minimizes it [the stigma]. Instead, it could just be widely accessible to anyone.”	
**Liked Educational experience**	
“A lot of my friends live in Florida and they’re all on PrEP. The first time I mentioned this to my doctor… he didn’t know anything about it. So I went back to him a month ago after talking with [the pharmacist], she said it was available, so I started PrEP three weeks ago now. I told him [doctor] I was tested and then he sent me for all the bloodwork again so then I started PrEP. But it all started through [the pharmacist]. So, going to a drug store, you have a more educated team telling you what’s available for preventive measures, so educate some more on that because I didn’t know it was available.”	
“Honestly if I had known I could have that kind of conversation with someone, I would have gotten this test years ago. He was just really good at explaining to me ‘ok, here’s what people think and here’s the reality and here’s what’s going to happen if there is a positive result’ and he just walked me through the whole thing…where all of the information I had before was just Internet stuff. If I had known that it could be that focused and calm a conversation, I would have done it earlier.”	
**Disliked POCT equipment**	
“When pharmacist was drawing the blood the use of the finger prick rather than a needle took a very long time to fill up the pipette. It didn’t hurt but it was uncomfortable.”	

Overall, participants felt strongly that the program should continue and HIV POCT should be routinely available in pharmacies. A minority of participants indicated their main reason for participating was to support the program in the hopes that it would become a routine testing option. Many participants specified there should be greater access to HIV POCT generally in their province. Many participants indicated they would be willing to pay for HIV testing at the pharmacy due to its convenience and out of desire to have access to HIV POCT. However, there was also a strong feeling that testing should be accessible to those who need it most, including those who may not have the means to pay. Participant quotes regarding their willingness to pay for HIV POCT are in Table 5.

**Table 5:** Quotes related to willingness to pay for testing

**Convenience and desire to access POCT**	
‘Oh yeah, for sure because I have to pay anywhere from $15 to $30 when I see [nurse practitioner] anyhow… I don’t mind slapping down $20 to go in right away where I don’t have to sit down for 3 hours. My time is worth money and $20 is nothing, I make more than that in 1 hour where I work. I mean it’s well worth it, right?”	
**Equity of access**	
”I think you’d have a lot more target audience, especially low income families, if it’s free. Sometimes these people are more at risk, people who are drug users are not going to go in and pay $25 for a test. But if [the government] covers it then you’ll have a lot more target audience to go after. So I don’t think it should have to be paid for. Financial barriers is one of the biggest barriers out there. I mean, I’d pay $10 to be honest but I know lots of people who wouldn’t.”	
“I would pay for the HIV testing only if there was a free service option available for those who cannot afford the test. If it’s being rolled-out in inner city communities, there should be a payment system whereby those who can afford it will pay a price, which may help cover the costs of those who cannot.”	

All participants indicated they would get tested again at a pharmacy in future.

### Pharmacist perceptions of the testing program

Regarding capability, participating pharmacists felt the training program prepared them well for offering the HIV POCT program. They felt that role play exercises designed to practice delivery of bad news and the depth of information covered by the training program contributed to their confidence in communicating with patients to deliver the HIV POCT service. They also valued the feedback received from the study team on their performance during the mock run-through in their own pharmacies to help them prepare to deliver the program. A consistent theme was that pharmacists experienced an initial nervousness but growing confidence that developed with each test they performed.

Participating pharmacists also felt that their prior professional experience helped prepare them for this role as counsellor, as they routinely deal with unexpected and sensitive situations in their practice. Their main apprehension was around the possibility of having to deliver a reactive HIV POCT result each time they did a test. Pharmacists felt that delivering the results of HIV screening directly is more impactful than supporting a person who has already received a diagnosis from their doctor and has had some time to process the information. Pharmacist quotes regarding capability are in Table 6.

**Table 6:** Pharmacist quotes regarding Capability

**Benefits of training**	
“It definitely does help to have that support available to receive feedbacks when its needed… support is basically a big thing for me and you guys being there to provide that training session and just giving that feedback right on the spot where you guys even said okay, we’ll stop the interaction, here’s what I think, I think those are, some of those feedbacks you guys gave me I still remember them so, definitely helps to approach the training session in that manner.”	
**Benefits of previous practice experience**	
“I feel like as pharmacists we deal with stuff, you know death and everything, and when that happens [positive POCT result]…it didn’t happen to us, thankfully… but if it did, I felt ok to deal with it.”	
**Confidence to provide HIV POCT service**	
“As soon as you had that experience after a few [tests]… I was able to more confidently speak about the process and about HIV in general when I wasn’t so focused on doing this, and then this and then this, I was able to kind of make it more fluid interaction and it was then that people opened up a lot more.”	
**Apprehension of delivering a positive HIV POCT result**	
“This was different because there was that looming potential for something to happen right then and there that could change somebody’s life and just knowing that you might be the one to have to deliver the news was, I think just another added stressor”	

Regarding opportunity, participating pharmacists felt the most important factor supporting the program was the clear linkage to care plan established for each pharmacy. Pharmacists routinely collaborate with other health professionals to provide care but knowing where and who specifically to contact to refer the patient in the event of a reactive test was critical. The pharmacists indicated they used the study tools (e.g. step-by-step flowchart, patient counselling handout) with every test performed and these were particularly helpful.

Overall, the pharmacists in this study felt the HIV testing program model worked well in their pharmacies. Logistic challenges related primarily to the use of testing pipettes and the paperwork required by the study. The testing pipettes did not readily collect the finger-prick blood sample, and this was felt to be the rate-limiting step in the testing process with many participants. The participant consent form and workload documentation were felt to be onerous and interrupted the flow or rapport with participants, and pharmacists felt these could be condensed outside of a research setting to be more practical.

Workflow considerations including whether to offer testing by drop-in or by appointment, how to determine designated testing hours (ie. day vs. evenings, weekends), staffing coverage, staff buy-in and support were felt to be significant. Specifically, participating pharmacists felt that participants preferred the ability to drop-in during advertised testing hours over having to make an appointment, and in several cases, people made appointments but did not show up. However, in the pharmacies where drop-in testing was offered, there were many days when there were no tests performed, which posed challenges for planning pharmacist schedules. Pharmacists felt it was important to ensure there was adequate staffing to support the program, including having multiple pharmacists trained to do testing to permit continuity of service. Lack of remuneration was felt to be the major challenge to the scale-up and sustainability of the HIV POCT program. Given the unpredictable and erratic nature of offering the testing service, remuneration and business models must consider how this successful testing model can be sustained. One pharmacist owner indicated she would consider hiring a “floater” pharmacist 1 day per week who would just do non-dispensing services such as medication reviews in addition to HIV POCT as a strategy to sustain the program, but remuneration is necessary. Pharmacist quotes regarding opportunity are in Table 7.

**Table 7:** Pharmacist quotes regarding Opportunity

**Importance of clear linkage to care plans**	
“If we provide this screening test and it came back positive, you know I think it’s important to know how we can collaborate with other healthcare professionals to follow up with the care, and obviously with this research project I think there was a really good system in place to ensure that had someone received the news that they might potentially be positive… that we can refer them to the designated nurse as well as the infectious disease specialist so that there is follow up.”	
**Usefulness of study tools and aids**	
“Yeah, I have [the flowchart] out every time in case I…sometimes the patient would start talking too and it would put you off track so then you’d just look to make sure you were going over everything.	
**Procedural challenges**	
“I just couldn’t get the blood to flow quickly enough [to fill the pipette] with one patient”	
**Pharmacy workflow considerations**	
“I think we were fortunate to be able to provide this service even though it did affect our operation a bit we had the luxury of having overlaps, that we were able to have pharmacist in the dispensary while one of us could step off for even half an hour, just to focus our time with the patient. I could see that being a huge challenge if it was a dispensary with one pharmacist.”	
**Need for remuneration**	
“I think for me that’s about the only disadvantage – being able to allocate resources properly as a business. To provide a service like this needs to be justifiable and as a result the remuneration model is imperative… it’s just how do you remove somebody from the workflow, justify whatever costs that incurs just in man hours in putting that pharmacist into that room for half an hour with the patient, the cost associated with removing them from workflow and the additional work that piles up as a result of that.”	

Regarding motivation, participating pharmacists felt strongly that offering HIV POCT was part of their professional role and identity. They perceived a value to the community by making HIV testing more accessible, particularly to those who might not otherwise get tested. Establishing professional relationships with participants and preventing unnecessary physician or emergency room visits were identified as motivating factors to providing pharmacist testing. Some felt their scope of practice could be broadened to order confirmatory HIV bloodwork if this became a regular service. Pharmacists felt excited and eager to offer HIV POCT in future. They described how they established good relationships with participants, and many have returned to become regular clients of the pharmacy as result of offering the testing program. Pharmacist quotes regarding motivation are in Table 8.

**Table 8:** Pharmacist quotes regarding Motivation

**Professional role and satisfaction**	
“Accessibility is really a big thing, like lots of people don’t have family doctors so they don’t know where to go to like get a lab [requisition] for a test so being able to just walk in to a pharmacy would be awesome to just get the test done right away… cause pharmacists like, there’s ample of us everywhere.”	
“I actually felt bad when the study ended and I had to send them somewhere else to get their testing.”	
**Important service**	
“[HIV POCT] just gave them a huge sense of relief, at least from the screening point of view that they knew their status or at least got a result immediately so … there is definitely a lot of value that I kind of learned from through the patients eyes… of this tool and just being accessible for the community”	
**Need for staff support**	
“When a pharmacist is doing testing in the private room with the client, the remaining staff have to cover off that pharmacist’s job in the dispensary, which creates more work. This might lead to resentment and may contribute to subtle negative feelings that could be picked up by clients and create a less than welcoming atmosphere if the entire team is not supportive of offering a testing program.”	

### Implementation of the HIV POCT program

The engagement of advisory committees in each province were critical to the success of this study to provide input into the study design, particularly linkage to care options and support services in each community. We engaged provincial public health laboratory officials early on to determine pharmacist tester competency and quality assurance needs. Their involvement informed the development of a robust training and quality assurance plan, and alleviated concerns regarding pharmacists performing HIV POCT in provinces where standards of practice were not yet developed to support this role. Pharmacy regulatory bodies in each province provided guidance to ensure pharmacists were practicing within their scope. Public health nurses and provincial HIV teams provided practical guidance to pharmacists on counselling during the testing program and served as a support throughout the study as needed. Community organizations were drivers of the program promotion and referred participants for testing.

Overall the HIV POCT program worked well in the pharmacies; however, some challenges were observed by the research team. The two rural pharmacy sites each had only one pharmacist trained to offer testing; in AB, this pharmacist moved to another province, which resulted in losing this study site early on in the study. Some pharmacies adjusted their advertised testing hours to reflect demand and the ability to offer consistent testing services after the first month of the study. Pharmacies who offered testing by appointment experienced some no-shows, which impacted staffing and workload to a minor degree. The urban pharmacies were specifically selected based on their location in an attempt to engage people who use drugs and those who engage in sex work for testing. Despite this, few participants reported these risk behaviours, raising questions as to whether the pharmacy HIV POCT program was not appealing or different promotion or engagement is necessary to reach these individuals. The location of one pharmacy was felt to be undesirable by some participants who reported feeling unsafe when travelling to the pharmacy, though they reported the pharmacy itself was a safe environment. However, it is not known the degree to which this issue may have influenced others to not seek testing as overall testing numbers were lower than anticipated at this location.

Maintaining the high level of promotion of the testing program over the course of the study was a challenge. Nearly half of the HIV POCTs were conducted in the first 6 weeks of the study when there were many stories about the study on the radio, and in television and print media. Spikes in testing correlated with media promotion throughout the study. Advertising on Grindr resulted in additional boosts in testing for one rural pharmacy site in particular (and was reported to be how the participant with the reactive test result heard about the program); however the costs of advertising were prohibitive to long-term use.

Based on observations throughout the study, review of pharmacy work logs, and feedback from the focus groups, program fidelity was high. However, participating pharmacists indicated they found the consent process cumbersome and too long, and they did not record the time spent on each aspect of the testing procedure as requested. They were confident in the measurement of total time spent with each participant but were more likely to estimate the proportion of time spent on each part of the testing process. They used the counselling handout consistently and only modified their counselling if the participant demonstrated knowledge of the points discussed already. Pharmacists indicated they adapted their time spent on some activities (e.g. consent) as the study progressed and they became more experienced. However, pharmacist experience did not impact time spent on counselling; rather, individual participant’s knowledge needs directed the depth and time spent on education. All pharmacists passed the quality assurance assessments and there were no indeterminate or invalid test results. Participants were accepting of the testing process with no refusals to provide information, though some questioned the purpose of collecting their health card number and several commented that the consent form was lengthy. All participants tested completed pre-test questionnaires, and 121/123 (98.4%) completed at least part of the post-test questionnaire, as requested.

## Discussion

Through the APPROACH study we were able to develop a pharmacist-provided HIV POCT program that was acceptable to clients and pharmacists. Of the participants tested during the pilot a high percentage of people identified as having never been tested for HIV before and were at an increased risk of having an undiagnosed HIV infection, which is consistent with studies of pharmacy-based HIV testing in other countries [21–24]. Although the focus of our pilot was to develop and assess a pharmacy-based testing program for scale up and future evaluation, we found one new case of HIV infection in the rural area of a province felt to have a low prevalence of HIV infection. Offering HIV testing through pharmacies, particularly in rural and remote areas where limited testing options exist, may be an effective way to reach the undiagnosed and successfully link individuals to follow up care.

Linkage to care plans for people who test HIV positive on both a POCT screening test and subsequent confirmatory testing is critical. A study involving Walgreens pharmacies in Virginia, United States found that 86.7% of participants with reactive screening tests received confirmatory testing, and of those with positive confirmatory test results, 84.6% were linked to care [22]. The pharmacies in the Walgreens study utilized a multistep linkage model which required the pharmacist and participant to call a partner organization or telephone support line to arrange confirmatory testing; linkage to care was made through the partner organization after the confirmatory test result was returned. Our goal was 100% linkage to care for participants with a reactive POCT result. To anticipate and mitigate any challenges with linkage to care, we engaged with our provincial advisory committee stakeholders to identify existing resources in each province that could facilitate a simple and direct linkage to care pathway. While we had only one participant with a reactive test result who went through our process, moving forward we believe this direct link by the pharmacist to send participants for confirmatory testing and reporting of those results through existing public health systems is both sustainable and less likely to result in losing people to follow up. However, it took considerable consultation and planning before implementing the testing program to establish this linkage to care plan, which is something pharmacists planning to offer HIV POCT services should consider.

The high degree of client acceptability in our study suggests that a pharmacist-provided HIV POCT program can overcome many barriers associated with HIV testing. Clients liked the privacy and discretion provided in the pharmacy, and the pharmacy venue was felt to normalize the testing experience. Many participants indicated they preferred going to the pharmacy over a sexual health clinic or requesting testing from their family doctor, attesting to the need for a variety of testing options that appeal to different people since barriers are different for everyone. The program had other important public health benefits in addition to helping people learn their HIV status, including increased awareness of the need for additional STBBI testing and education about preventative measures including the use of pre-exposure prophylaxis (PrEP). Increasing STBBI testing with subsequent linkage to care and treatment, and reducing the risk of new infections are important public health goals that may be realized through scale up of pharmacy-based testing programs.

For an HIV POCT program to be successfully scaled up, implementation barriers and supports must be considered. A recent review of implementation factors necessary to support a variety of pharmacist services, including HIV testing was published [25]. Consistent with our study findings, most challenges and opportunities to offering HIV POCT were related to “inner setting” factors such as pharmacist training, staff and physical supports, and tensions regarding offering a variety of pharmacy services with limited resources. Enablers to providing an HIV testing service included having a public health orientation, being committed to offering preventative services, and having partnerships with health departments [25]. In our study, pharmacists and participants found the consent documentation (required by the research ethics committee) to be onerous and time-consuming, taking up to 10 min to complete each time. While 30 min to complete an HIV POCT is comparable to the experience in other studies [21], outside of a research context the client consent process could be streamlined similar to other pharmacy services such as influenza vaccination, to reduce time and documentation demands but remain in accordance with regulatory standards. Scaling up and sustaining a pharmacy-based HIV POCT program for the long term would benefit from integration with provincial or regional testing programs and services already in existence. Development of pharmacy standards for HIV POCT, competency certification, and resources to identify and support linkages to care for confirmatory testing and care for clients would ensure that testing services are offered in an appropriate and consistent manner. Integration of pharmacy HIV POCT services with existing provincial or regional public health and communicable diseases services could achieve cost savings and efficiencies through bulk purchasing and central distribution of POCT kits to minimize wastage by using them within the expiry dates and streamline quality assurance processes for testing, identification and reporting of any issues related to performance of POCT kits.

Moving forward the main challenges of time and remuneration must be addressed in order to fully assess whether the pharmacist-provided HIV POCT program is sustainable. These barriers are not unique to the pharmacy environment as they have been cited as barriers for physicians and other health providers to offering HIV testing in their practice settings [26–28]. However, pharmacists have unique contextual challenges in that they practice in a retail environment with product-based incentive structures rather than individual billing numbers for provision of professional services, and they have to integrate client-focused services within a dispensary workflow [25]. If remuneration models can be established, the pharmacy-based HIV POCT model is a feasible, highly desirable testing option to reach those who may be at risk and have never been tested. The model could further be adapted to offer testing for other STBBIs, in addition to HIV [29].

There are several limitations of this study. The most notable limitation is the low number of tests performed over the study period. The aim of the study was to test a minimum of 30 participants over the 6-month pilot in order to inform any modifications necessary prior to a broader scale implementation of the program, thus the study was not designed or powered to demonstrate effectiveness, sustainability or cost-effectiveness of the testing model [14]. There are a number of factors which may have limited the number of tests performed. Participating pharmacies had only one or two trained pharmacist testers per store, which limited the ability to offer the service during usual hours of operation. Sites had limited testing hours; some offered testing just 1 day per week and testing was not routinely available on nights and weekends, which reduced access for clients who could not attend during these hours. Clients also reported a preference for drop-in over appointment-based testing, the latter being the only option for some pharmacies. In addition, the loss of one of the rural testing sites early in the study and the location of an urban pharmacy in the inner city affected the numbers tested in one province. Our small sample size could lead to an over-representation of acceptability and satisfaction with the program, and may not be generalizable outside of the Canadian context; however, strengths of this research include the use of a hybrid implementation-effectiveness design and mixed methods to evaluate client acceptability.

Future scale-up of the pharmacy testing model can address these limitations by ensuring multiple pharmacists are trained and available to offer testing on a more consistent and regular basis during usual pharmacy business hours. Consistent promotion of testing services, offering testing to clients when they come into the pharmacy, and budgeting for the strategic use of geosocial and dating app advertisements may increase testing rates based on our experience as we saw significantly higher uptake during times when testing was advertised through media or on Grindr.

The measurement of success of an HIV testing program should consider not only the number of tests performed, but importantly, reaching the undiagnosed. With a population of approximately 525,000 people, NL is considered a low HIV prevalence province (HIV diagnosis rate in Atlantic region = 2.8/100,000 people) [30]. However, as HIV testing rates are low, particularly in rural areas where there is a lack of sexual health services and limited access to family physicians, there is a concern that we may be missing diagnoses. Nearly one-third (32.1%) of NL participants in APPROACH indicated they had not had an HIV test previously or were uncertain. And while the rural NL site completed a small number of tests overall, they did find one new case of HIV infection. This individual belonged to a priority population but was unable to access testing as he did not have a family doctor or access to sexual health services. The pharmacy offered easy access to rapid test results, and he was directly linked with follow up care. Increasing access to testing in an acceptable manner to all individuals at risk of HIV infection is important to reach the remaining 14% of the Canadian population who are unaware of their status, and achieve Canada’s 90–90-90 targets [31].

Building on the success and the lessons learned from the APPROACH study, future research will assess the scale-up, effectiveness, and cost-effectiveness of the pharmacy-based testing program in three Canadian provinces. Enhancement of the pharmacy testing program in the next study, APPROACH 2.0, will include testing for hepatitis C and other STBBIs, in addition to HIV.

## Conclusion

The APPROACH study demonstrated that a pharmacy-based testing program for HIV POCT is feasible and highly acceptable to participants and pharmacists. Findings from this research are useful to inform enhancements and scale-up of this model. Future research will evaluate the effectiveness and cost-effectiveness of this approach in reaching people at high risk of HIV.

## Data Availability

The datasets used and/or analyzed during the current study are available from the corresponding author on reasonable request.

## Abbreviations

*AB*: Alberta
*ANOVA*: Analysis of variance
*APPROACH*: Adaptation of POCT for Pharmacies to Reduce risk and Optimize Access to Care in HIV
*HIV*: Human Immunodeficiency Virus
*MANOVA*: Multivariate analysis of variance
*POCT*: Point-of-care testing
*PrEP*: Pre-exposure prophylaxis
*MSM*: Men who have sex with men
*NL*: Newfoundland and Labrador
*RA*: Research assistant
*STBBI*: Sexually transmitted and bloodborne infections

## Footnotes

1 APPROACH stands for Adaptation of POCT for Pharmacies to Reduce risk and Optimize Access to Care in HIV

## References

[1] Joint United Nations Programme on HIV/AIDS, 90–90-90 (2014). An ambitious treatment target to help end the AIDS epidemic.

[2] Marks G, Crepaz N, Janssen RS (2006). Estimating sexual transmission of HIV from persons aware and unaware that they are infected with the virus in the USA. AIDS.

[3] Marks G, Crepax N, Senterfitt JW, Janssen RS (2005). Meta-analysis of high-risk sexual behavior in persons aware and unaware they are infected with HIV in the United States: implications for HIV prevention programs. J Acquir Immune Defic Syndr.

[4] Traversy GP, Austin T, Ha S (2015). An overview of recent evidence on barriers and faciliators to HIV testing. CCDR.

[5] Spielberg F, Branson BM, Goldbaum GM (2003). Overcoming barriers to HIV testing: preferences for new strategies among clients of a needle exchange, a sexually transmitted disease clinic, and sex venues for men who have sex with men. J Acquir Immune Def Syndr.

[6] Molitor F, Walsh RM, Leigh JP (2002). Determinants of longer time from HIV result to enrolment in publicly funded care and treatment in California by race/ethnicity and behavioral risk. AIDS Patient Care STDs.

[7] REACH 2.0. CIHR CBR Collaborative (2015). Rapid HIV point-of-care testing in non-urban settings: a scoping review.

[8] Stevens W, Gous N, Ford N, Scott LE (2014). Feasibility of HIV point-of-care test for resource-limited settings: challenges and solutions. BMC Med.

[9] Guenter D, Greer J, Barbara A (2008). Rapid point-of-care HIV testing in community-based anonymous testing program: a valuable alternative to conventional testing. AIDS Patient Care STDs.

[10] Lewis NM, Gahagan JC, Stein C (2013). Preferences for rapid point-of-care HIV testing in Nova Scotia, Canada. Sex Health.

[11] Gahagan J, Minichiello A, Swab M (2019). HIV point-of-care testing in non-urban settings: a coping review. Can J Human Sexuality.

[12] Minichiello A, Swab M, Chongo M (2017). HIV point-of-care testing in Canadian settings: a scoping review. Front Public Health.

[13] Curran GM, Bauer M, Mittman B (2012). Effectiveness-implementation hybrid designs: combining elements of clinical effectiveness and implementation research to enhance public health impact. Med Care.

[14] Kielly J, Kelly DV, Hughes C (2018). Adaptation of POCT for pharmacies to reduce risk and optimize access to care in HIV, the APPROACH study protocol: examining acceptability and feasibility. Pilot and Feasibility Studies.

[15] Grindr. https://www.grindr.com/about/ (2019). Accessed 5 Mar 2019.

[16] Health Agency of Canada. Human immunodeficiency virus HIV Screening and Testing Guide. Available at: https://www.canada.ca/en/public-health/services/hiv-aids/hiv-screening-testing-guide.html. Accessed 2 Mar 2019.

[17] Haukoos JS, Lyons MS, Lindsell CJ (2012). Derivation and validation of the Denver human immunodeficiency virus (HIV) risk score for targeted HIV screening. Am J Epi.

[18] Haukoos JS, Hopkins E, Bender B (2013). Comparison of enhanced targeted rapid HIV screening using the Denver HIV risk score to nontargeted rapid HIV screening in the emergency department. Ann Emerg Med.

[19] Castleberry A, Nolen A (2018). Thematic analysis of qualitative research data: is it as easy as it sounds?. Curr Pharm Teach Learn.

[20] Mitchie S, van Stralen MM, West R (2011). The behaviour change wheel: a new method for characterising and designing behaviour change interventions. Implement Sci.

[21] Darin KM, Klepser ME, Klepser DE (2015). Pharmacist-provided rapid HIV testing in two community pharmacies. J Am Pharm Assoc.

[22] Collins B, Bronson H, Elamin F, et al. The “No wrong door” approach to HIV testing: Results from a statewide retail pharmacy-based HIV testing program in Virginia, 2016-2016. Pub Health Reports. 2018;133(Suppl 2):34S–42S. 10.1177/0033354918801026 PMC6262519 30457955

[23] Fernandez-Balbuena S, Belza MJ, Zulaica D, et al. Widening the access to HIV testing: the contribution of three in-pharmacy testing programmes in Spain. PLOS ONE. 10(8):e0134631. 10.1371/journal.pone.0134631. 10.1371/journal.pone.0134631 PMC4527698 26247367

[24] Amesty S, Crawford ND, Nandi V (2015). Evaluation of pharmacy-based HIV testing in a high-risk New York City Community. AIDS Patient Care STDs.

[25] Shoemaker SJ, Curran GM, Swan H (2017). Application of the consolidation framework for implementation research to community pharmacy; a framework for implementation research on pharmacy services. Res Social Admin Pharm.

[26] Fielden S, Lindegger M, Pedersen H, et al. Evaluation findings from the pilot phase of BC’s provincial point of care HIV testing program: the first 18 months. Available at: http://www.bccdc.ca/resource-gallery/Documents/Statistics%20and%20Research/Statistics%20and%20Reports/STI/CPS_POC_Program_Eval_Report_20130823.pdf. Accessed Feb 16, 2019.

[27] Hidnocha S, Charlton T, Rayment M, Theobald N (2013). Feasibility and acceptability of routine human immunodeficiency virus testing in general practice: your views. Prim Health Care Res Dev.

[28] Milligan R, Obasi A (2014). Attitudes of general practitioners to the introduction of routine human immunodeficiency virus testing in United Kingdom primary care. HIV Med.

[29] Wood H, Gudka S (2018). Pharmacist-led screening in sexually transmitted infections: current perspectives. Integr Pharm Res Pract.

[30] Haddad N, Robert A, Weeks A (2019). HIV in Canada – surveillance report, 2019. Can Commun Dis Rep.

[31] Public Health Agency of Canada (2018). Summary: Estimates of HIV incidence, prevalence and Canada’s progress on meeting the 90–90-90 HIV targets, 2016. Report.

